# Retinal Pigment Epithelium-Secreted VEGF-A Induces Alpha-2-Macroglobulin Expression in Endothelial Cells

**DOI:** 10.3390/cells11192975

**Published:** 2022-09-24

**Authors:** Guillermo L. Lehmann, Michael Ginsberg, Daniel J. Nolan, Cristina Rodríguez, José Martínez-González, Shemin Zeng, Andrew P. Voigt, Robert F. Mullins, Shahin Rafii, Enrique Rodriguez-Boulan, Ignacio Benedicto

**Affiliations:** 1 Margaret Dyson Vision Research Institute, Department of Ophthalmology, Weill Cornell Medicine, New York, NY 10065, USA; 2 Regeneron Pharmaceuticals, Inc., Tarrytown, NY 10591, USA; 3 Angiocrine Bioscience, Inc., San Diego, CA 92121, USA; 4 Institut de Recerca Hospital de la Santa Creu i Sant Pau, 08041 Barcelona, Spain; 5 Institut d’Investigació Biomèdica Sant Pau (IIB SANT PAU), 08041 Barcelona, Spain; 6 CIBER de Enfermedades Cardiovasculares (CIBERCV), Instituto de Salud Carlos III, 28029 Madrid, Spain; 7 Instituto de Investigaciones Biomédicas de Barcelona-Consejo Superior de Investigaciones Científicas (IIBB-CSIC), 08036 Barcelona, Spain; 8 Institute for Vision Research, Department of Ophthalmology and Visual Sciences, The University of Iowa, Iowa City, IA 52246, USA; 9 Ansary Stem Cell Institute, Department of Medicine, Division of Regenerative Medicine, Weill Cornell Medicine, New York, NY 10065, USA; 10 Departamento de Inmunología, Oftalmología y ORL, Facultad de Medicina, Universidad Complutense de Madrid, 28040 Madrid, Spain; 11 Centro Nacional de Investigaciones Cardiovasculares (CNIC), 28029 Madrid, Spain

**Keywords:** Alpha-2-macroglobulin (A2M), retinal pigment epithelium (RPE), vascular endothelial growth factor-A (VEGF-A), endothelial cells (ECs), extracellular matrix (ECM), matrix metalloproteinase-2 (MMP-2), protease inhibitor, age-related macular degeneration (AMD), Bruch’s membrane

## Abstract

Alpha-2-macroglobulin (A2M) is a protease inhibitor that regulates extracellular matrix (ECM) stability and turnover. Here, we show that A2M is expressed by endothelial cells (ECs) from human eye choroid. We demonstrate that retinal pigment epithelium (RPE)-conditioned medium induces A2M expression specifically in ECs. Experiments using chemical inhibitors, blocking antibodies, and recombinant proteins revealed a key role of VEGF-A in RPE-mediated A2M induction in ECs. Furthermore, incubation of ECs with RPE-conditioned medium reduces matrix metalloproteinase-2 gelatinase activity of culture supernatants, which is partially restored after A2M knockdown in ECs. We propose that dysfunctional RPE or choroidal blood vessels, as observed in retinal diseases such as age-related macular degeneration, may disrupt the crosstalk mechanism we describe here leading to alterations in the homeostasis of choroidal ECM, Bruch’s membrane and visual function.

## 1. Introduction

Photoreceptors are the retinal cells that capture incoming photons in the eye and transform them into electrical pulses that ultimately lead to visual perception. Photoreceptor function depends on the retinal pigment epithelium (RPE), a cell layer adjacent to the photoreceptors that enables visual function by recycling visual cycle components, eliminating retinal waste products and regulating ion and fluid flux between the subretinal space and the choroid [1]. The RPE sits on top of Bruch’s membrane, a complex extracellular matrix (ECM) compartment that separates the RPE from the underlying choroidal capillaries. Blood supplied by the choroidal circulation is the main source of oxygen and nutrients for RPE and photoreceptors and the main evacuation route for retinal waste [2]. Thus, choroidal perfusion is essential for retinal homeostasis and visual function. However, recent studies have shown that microvascular endothelial cells (ECs) are not just passive conduits for delivering blood. Rather, ECs are tissue-specific and regulate organ homeostasis and regeneration by providing highly specialized sets of angiocrine factors at different body locations [3].

EC heterogeneity appears to be the result of local cues provided by parenchymal cells, as stem cell-derived generic ECs were shown to acquire tissue-specific features after transplantation and engraftment in different organs [4]. The viability of choroidal capillaries is known to depend on RPE-secreted vascular endothelial growth factor-A (VEGF-A) [5,6]; however, it remains largely unknown how RPE-secreted VEGF-A and other factors regulate choroid EC gene expression and function. In a previous study, we demonstrated RPE-choroid EC crosstalk in the opposite direction, as we showed that EC-secreted factors modulate RPE basement membrane, tight junctions, and barrier function [7]. We reported the transcriptomes of developing (P5) and adult (P30) mouse choroid ECs; they revealed that, as the outer retina undergoes terminal differentiation, choroid ECs become enriched in transcripts encoding ECM-related proteins [7]. Our results suggest an important role of choroid ECs in the assembly and homeostasis of choroidal ECM and Bruch’s membrane.

Age-related macular degeneration (AMD) affects 25% of people over 80 years old, making it a leading cause of blindness in developed countries [8]. Although the etiology and pathogenesis of AMD are only emerging, genome-wide association studies have shown a significant correlation between AMD and the presence of genetic variants near ECM-related genes such as *TIMP3*, *COL8A1*, *COL4A3*, *MMP19* and *MMP9* [9]. Moreover, the abundance of protease inhibitors such as TIMP3 or serine protease inhibitors (SERPINs) has been shown to be altered in the choroid and Bruch’s membrane of AMD patients [10,11]. Furthermore, AMD progression induces choroidal thinning [11], which may be related to the alteration of choroidal ECM homeostasis. Indeed, microarray and proteome studies suggest that AMD induces changes in RPE/choroid expression of factors involved in ECM remodeling [12,13]. All these pieces of evidence strongly suggest that proper control of ECM turnover is essential for the correct maintenance of Bruch’s membrane and choroidal stroma and that perturbation of the fine-tuned balance between choroidal proteases and their inhibitors may contribute to AMD pathogenesis. 

Vertebrate alpha-2-macroglobulin (A2M) is a tetrameric 720 kDa secreted glycoprotein that belongs to the alpha-macroglobulin family, which also includes pregnancy zone protein (PZP) and complement factors C3, C4 and C5 [14,15]. A main feature of A2M is its ability to inhibit a broad spectrum of proteases, including ECM-remodeling matrix metalloproteinases [16,17]. The inhibitory mechanism involves protease-mediated cleavage of a ‘bait region’ of A2M, which triggers a conformational change that traps the protease and hinders its access to other targets. In addition, A2M cleavage exposes a binding site for LRP1, the main A2M receptor, enabling the endocytosis and clearance of A2M -protease complexes from the circulation mainly by hepatocytes, or by other LRP1-expressing cells such as tissue macrophages [18,19]. Here, we analyzed previously published single cell (sc)RNAseq data from human RPE/choroid tissue to show that choroidal ECs express A2M. Mechanistic studies revealed that RPE-secreted VEGF-A induces A2M expression in ECs, which in turn reduces matrix metalloproteinase-2 (MMP-2) gelatinase activity of culture supernatants. We discuss the potential implications of such RPE-choroid EC crosstalk in health and disease.

## 2. Materials and Methods

### 2.1. Human RPE/Choroid scRNAseq Analysis

Previously published scRNAseq data from human RPE/choroid and retinal tissue [20,21,22,23,24] were analyzed using Spectacle software [25]. Qualitative analysis was carried out using Spectacle’s dataset ‘all_retina_rpe_chor’, containing data from 13 donors, 32 libraries, and >90,000 sequenced cells. Differential gene expression analysis was carried out using the datasets ‘RPE_choroid_unselected’ (3 donors, paired macular and peripheral RPE/choroid tissue) and ‘RPE_choroid_CD31_selected’ (4 donors, paired macular and peripheral RPE/choroid tissue, sequencing carried out after enrichment of CD31^+^ ECs). 

### 2.2. Cell Culture

Human fetal RPE (hfRPE) cells from donors at 16–18 weeks of gestation were kindly provided by S.S. Miller (NIH) and cultured at 37 °C, 5% CO_2_ in RPE medium [7,26]: MEM, α modification containing N1 supplement 100×-Solution, hydrocortisone (20 µg L^−1^), taurine (250 mg L^−1^), triiodo-thyronin (0.013 µg L^−1^) (Sigma-Aldrich, St. Louis, MO, USA), Penicillin-Streptomycin 50×-solution (Corning Inc., Corning, NY, USA), 5% or 1% fetal bovine serum (FBS) (see Section 2.3 below), Glutamax 100×-solution and MEM non-essential amino acids 100×-solution (Life Technologies, Carlsbad, CA, USA). Cells were used in passage 0 or 1. 293T and HeLa cells (ATCC, Manassas, VA, USA) were cultured in DMEM (Corning Inc.), 10% FBS (Life Technologies) and Penicillin-Streptomycin 50× Solution (Corning Inc.). E4ORF1-expressing HUVECs [7,27] (provided by Angiocrine Bioscience, San Diego, CA, USA) were cultured at 37 °C, 5% CO_2_ in EC growth medium: HyClone M199 (Cytiva, Marlborough, MA, USA), 50 µg mL^−1^ endothelial cell supplement BT-203 (Alfa Aesar, Haverhill, MA, USA), 20% FBS (Life Technologies), Penicillin-Streptomycin 50×-solution (Corning Inc.), 10 mM HEPES (Invitrogen, Waltham, MA, USA), 50 µg mL^−1^ heparin (Sigma-Aldrich) and Glutamax 100×-solution (Life Technologies), and were used in all experiments unless otherwise indicated. E4ORF1 expression in ECs enables their survival and the maintenance of the endothelial phenotype in the absence of serum or any added factors [27]. Naïve HUVECs (Lonza, Basel, Switzerland) were used to test the effect of recombinant VEGF-A on A2M expression and in transient transfection assays. They were used between passages 2 and 4 and cultured at 37 °C, 5% CO_2_ in M199 supplemented with 20 mM HEPES pH 7.4, 2 mM glutamine, 1 mM pyruvate, 0.1 mg mL^−1^ streptomycin, 100 U mL^−1^ penicillin (Gibco, Thermo Fisher Scientific, Waltham, MA, USA), 30 µg mL^−1^ endothelial growth factor supplement, 100 µg mL^−1^ heparin (Sigma-Aldrich) and 20% FBS (Biological Industries, Beit Haemek, Israel) as described [28].

### 2.3. Conditioned Medium and A2M Induction Assays

hfRPE cells were cultured as previously described [7]. Briefly, 2 × 10^5^ hfRPE cells were seeded on polyester membrane Transwell inserts (12 mm diameter, 0.4 μm pore; Corning Inc.) in RPE medium supplemented with 5% FBS and 10 μM Y-27632 (Tocris, Bristol, UK). After culturing cells for 3 days, media was switched to RPE medium supplemented with 1% FBS, which was changed every 3–4 days. Transepithelial electrical resistance (TER) was measured in triplicate with an EVOM Voltohmeter (World Precision Instruments, Sarasota, FL, USA) before changing media, and TER values were expressed in ohms (Ω) cm^2^ after background subtraction. Unless otherwise indicated, hfRPE were cultured for 4–5 weeks before collecting conditioned media from the basolateral chamber 3–4 days after the last media change. Conditioned media were aliquoted and stored at −80 °C until used. ECs, 293T and HeLa cells were cultured in 48-well plates until confluency, when their regular growth media were replaced by undiluted 1% FBS RPE medium (mock) or hfRPE conditioned media. Where indicated, RPE conditioned medium was serially diluted in 1% FBS RPE medium or boiled for 5 min before induction. SU5614 (Sigma-Aldrich), control IgG (cat. AB-108-C, R&D Systems, Minneapolis, MN, USA) or anti-VEGF-A_165_ antibody (cat. AF-293-NA, R&D Systems) were added to the conditioned media as indicated. Cells and supernatants were processed at different time points as indicated.

### 2.4. Real-Time PCR

RNA was extracted with the RNeasy Mini Kit (Qiagen, Hilden, Germany). cDNA was prepared with the High Capacity cDNA Reverse Transcription Kit (Life Technologies, Carlsbad, CA, USA) and real time PCR was carried out in a StepOnePlus Real-Time PCR System (Life Technologies) using SYBR Select Master Mix (Life Technologies) and the following primer pairs: human *A2M* (NM_000014, 5′-gtctggttcttctcctcttg-3′ and 5′-gcacttacagtcactgtctc-3′), and human *KDR* (VEGFR2) (NM_002253, 5′-tggcctcggtcatttatgtc-3′ and 5′-gcacaaagtgacacgttgag-3′). Human *GAPDH* (NM_002046, 5′-ggctggggctcatttgcaggg-3′ and 5′-tgaccttggccaggggtgct-3′) was used as loading control and relative expression values were calculated by the 2^−ΔΔCt^ method.

### 2.5. Western Blot

Culture supernatants were cleared by centrifugation (400× *g*, 3 min) and combined with 6× Laemmli buffer plus 2% β-mercaptoethanol. Equal volumes of culture supernatants from every tested condition were analyzed in each experiment. Cells were lysed in 1% Triton X-100, 0.1% SDS, 40 mM Tris-HCl pH 7.6, 150 mM NaCl, and protease inhibitor cocktail III (EMD Millipore, Merck, Darmstadt, Germany) for 15 min at 4 °C. Lysates were cleared by centrifugation (15,700× *g*, 5 min at 4 °C) and combined with 2× Laemmli buffer plus 2% β-mercaptoethanol. Equal volumes of cell lysates from every tested condition in each experiment were analyzed for GAPDH levels and used as a loading control proxy for A2M and MMP-2 levels in culture supernatants. Non-boiled samples were run in SDS-polyacrylamide gels and analyzed by Western blot using mouse anti-human A2M antibody (1:1000, clone 2D9, cat. ab36995, Abcam, Cambridge, UK) and rabbit polyclonal antibodies against human MMP-2 (1:1000, cat. 4022, Cell Signaling Technology, Danvers, MA, USA) and GAPDH (1:2000, cat. 2275-PC-100, Trevigen, Gaithersburg, MD, USA). The original, unmodified, and uncropped images from all Western blots presented are shown in Supplementary Figure S1.

### 2.6. ELISA

hfRPE cells were cultured as described above and basolateral conditioned media were collected after 1 and 4 weeks in culture, 4 days after last media change. VEGF-A levels were determined with the human VEGF Quantikine ELISA Kit (R&D Systems, Minneapolis, MN, USA) following the manufacturer’s recommendations.

### 2.7. Cell Viability Assays

ECs were incubated at 37 °C for 24 h in hfRPE conditioned media in the absence or presence of 2 µM SU5614 or 1 µg mL^−1^ antibodies as indicated, and 10% alamarBlue (Invitrogen, Waltham, MA, USA) was included for the last 90 min. Fluorescence was measured in a microplate reader (excitation 560 nm, emission 590 nm).

### 2.8. Transient Transfection and Luciferase Assays

Naïve HUVECs were transfected with a luciferase reporter construct corresponding to human *A2M* promoter (pGL3/hA2M-1999) [29] using Lipofectamine LTX with Plus Reagent (Invitrogen) according to the manufacturer’s protocol. Briefly, transient transfections were performed in subclonfluent cells seeded in 12 well plates using 1 µg of the luciferase reporter plasmid, 0.05 µg pRL-SV40 (Promega, Madison, WI, USA) as an internal control, 1 µL Plus Reagent and 4 µL Lipofectamine LTX Reagent per well. After incubating the cells with the DNA/liposome complexes for 8 h, cells were washed once with arrest medium containing 10% FBS (without heparin or endothelial growth factor), incubated in arrest medium overnight and treated with 50 ng mL^−1^ VEGF-A (R&D Systems) for 8 h. Firefly and renilla luciferase activities were determined in cell lysates using the Dual-Luciferase Reporter Assay System (Promega) and an Orion I luminometer (Berthold Detection Systems, Pforzheim, Germany). Results were expressed as the ratio of firefly to renilla activity. Parallel real-time PCR assays were carried out in untransfected cells in the same conditions to assess the induction of endogenous A2M by recombinant VEGF-A.

### 2.9. Zymography Assays

Cell culture supernatants were combined with 6× Laemmli buffer under non-reducing conditions and run on SDS-polyacrylamide gels embedded with 1 mg mL^−1^ gelatin (Sigma-Aldrich). Gels were washed in 2.5% Triton X-100 for 30 min at room temperature and the wash was repeated for a total of three times. Gels were rinsed twice in distilled water and incubated in 50 mM Tris-HCl pH 7.5, 10 mM CaCl_2_, and 200 mM NaCl overnight at 37 °C. Gels were stained 1 h at room temperature with 50% methanol, 10% acetic acid and 0.1% Coomassie blue, and washed in 50% methanol and 10% acetic acid several times until non-stained bands (corresponding to areas of gelatinolytic activity) were clearly visible. Gels were scanned and bands were quantified with ImageJ software (v1.53m, National Institutes of Health, Bethesda, MD, USA). ProMMP-2 activity was quantified in the same gels as MMP-2 activity and used for normalization. The original, unmodified, and uncropped images from all zymography assays presented are shown in Supplementary Figure S2.

### 2.10. Lentiviral Production and shRNA Assays

A pLKO.1-derived lentiviral shRNA construct targeting human A2M was obtained from Sigma Mission (TRCN0000006656). A non-targeting sequence (SHC016, Sigma-Aldrich) was cloned into pLKO.1 (Addgene) and used as control. Lentiviral particles were generated as previously described [27] and viral supernatants were concentrated 100-fold with the Lenti-X Concentrator (Clontech, Takara Bio USA Inc., San Jose, CA, USA) following the manufacturer’s instructions. For virus titration, RNA from viral preparations was isolated with the RNeasy Mini Kit (Qiagen), including an in-column DNase digestion step. After cDNA preparation, real time PCR was carried out using the HIV RRE-specific primers (5′-GTATAGTGCAGCAGCAGAAC-3′ and 5′-ACAGCAGTGGTGCAAATGAG-3′). Quantification was performed by interpolation into a standard curve. ECs were grown on 48 well plates until confluency and transduced with ~5 × 10^7^ viral genome equivalents/well in EC growth medium supplemented with 6 μg mL^−1^ polybrene (Sigma-Aldrich). After 72 h media were replaced by 1% FBS RPE medium (mock) or hfRPE conditioned media, and 72 h later cells and supernatants were processed for further analysis.

### 2.11. Statistical Analysis

All data are presented as mean + standard deviation. The number of biological replicates (*n*) is indicated in each figure legend. Statistical significance was calculated using two-tailed *t*-test or one-way ANOVA plus Bonferroni post hoc analysis (*, *p* < 0.05; **, *p* < 0.01; ***, *p* < 0.001) as indicated. No statistical method was used to predetermine sample size or to test for normality and variance homogeneity.

## 3. Results

### 3.1. A2M Expression Is Enriched in Human Choroidal and Retinal ECs

**Figure 1:**
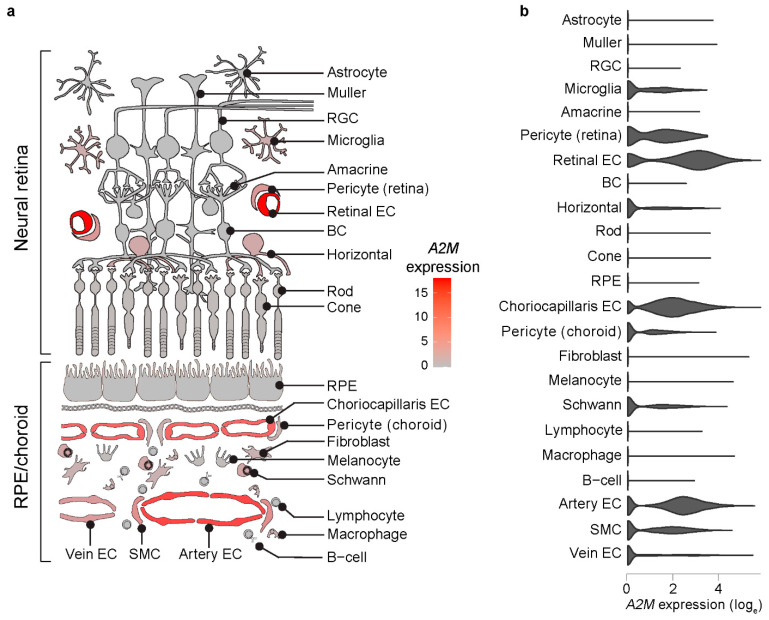
Enrichment of A2M expression in human choroidal and retinal ECs assessed by scRNAseq. Cartoon heatmap (**a**) and violin plots (**b**) depicting A2M expression across human RPE/choroid and retinal cells. RGC, retinal ganglion cell; BC, bipolar cell; RPE, retinal pigment epithelium; SMC, smooth muscle cell.

First, we explored A2M expression pattern in human RPE/choroid and retina by analyzing published scRNAseq data obtained from 13 donors [20,21,22,23,24] using Spectacle software [25] (v0.3.1, Institute for Vision Research, Iowa City, IA, USA). We found that A2M is primarily expressed in choroidal and retinal ECs (Figure 1). Focusing on RPE/choroid tissue, differential expression analysis showed a significant enrichment of A2M expression in choroidal ECs compared to the rest of RPE/choroid cell types (average log_e_ fold change = 0.59; adjusted *p*-value = 6.48 × 10^−29^). Within choroidal ECs, choriocapillaris and artery ECs showed significantly higher A2M expression than vein ECs (average log_e_ fold change = 0.78 and 0.62; adjusted *p*-value = 8.44 × 10^−89^ and 9.93 × 10^−188^, respectively). 

### 3.2. RPE Conditioned Medium Induces A2M Expression in ECs

**Figure 2:**
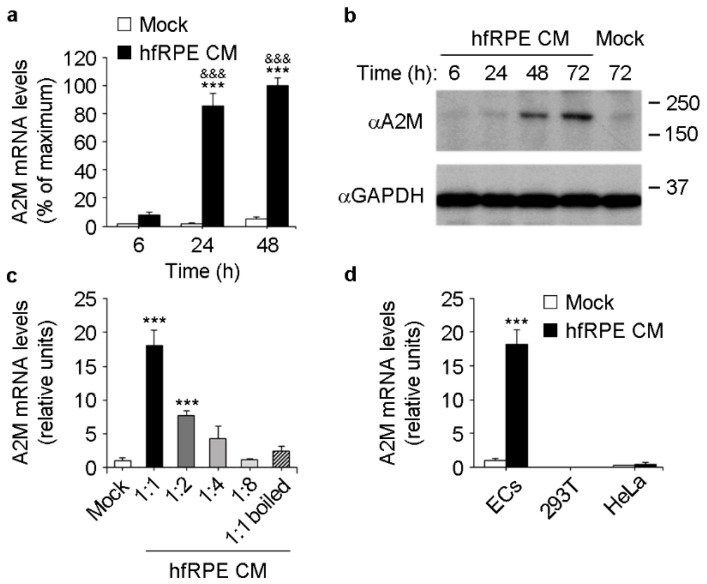
RPE conditioned medium induces A2M expression specifically in ECs. (**a**) Real-time PCR showing A2M mRNA levels in ECs after incubation with mock or hfRPE basolateral conditioned media (CM) for different times (*n* = 3, ANOVA). ***, mock vs. hfRPE at each time point; &&&, 24 h and 48 h hfRPE vs. 6 h hfRPE. (**b**) Representative Western blots (*n* = 3) showing A2M and GAPDH levels in culture supernatants and lysates, respectively, of ECs after incubation with mock or hfRPE basolateral conditioned media for different times. (**c**) Real-time PCR showing a dose-dependent effect of hfRPE basolateral conditioned media on A2M expression in ECs 24 h after induction. Numbers on the X-axis indicate the dilution factor. Note that A2M induction was impaired after heat denaturation (1:1 boiled) of the undiluted hfRPE conditioned media (*n* = 3, ANOVA). ***, 1:1 and 1:2 vs. mock. (**d**) Real-time PCR showing that induction of A2M expression by hfRPE basolateral conditioned media (24 h incubation) is specific for ECs (*n* = 3, *t*-test). ***, mock vs. hfRPE.

We hypothesized that RPE-derived factors may induce A2M expression in choroidal ECs. To test this hypothesis in vitro, we used basolateral conditioned media from primary, polarized human fetal RPE (hfRPE) cultured on Transwell inserts, and primary human umbilical vein ECs (HUVECs). ECs were incubated with mock or RPE basolateral conditioned medium for different times, after which RNA was extracted and culture supernatants were collected. Incubation of ECs with RPE conditioned medium induced a time-dependent increase in A2M mRNA levels (Figure 2a) as well as the accumulation of A2M in culture supernatants (Figure 2b). Induction of A2M expression was lost after heat denaturation of RPE conditioned medium (Figure 2c) and was dose-dependent, as serial dilution of RPE conditioned medium resulted in progressively reduced A2M induction (Figure 2c). Importantly, RPE conditioned medium did not induce A2M expression in the non-endothelial cell lines 293T and HeLa (Figure 2d). These data strongly suggest that polarized RPE secrete factors through the basolateral plasma membrane that induce A2M expression specifically in ECs. 

### 3.3. RPE-Secreted VEGF-A Induces A2M Expression in ECs

**Figure 3:**
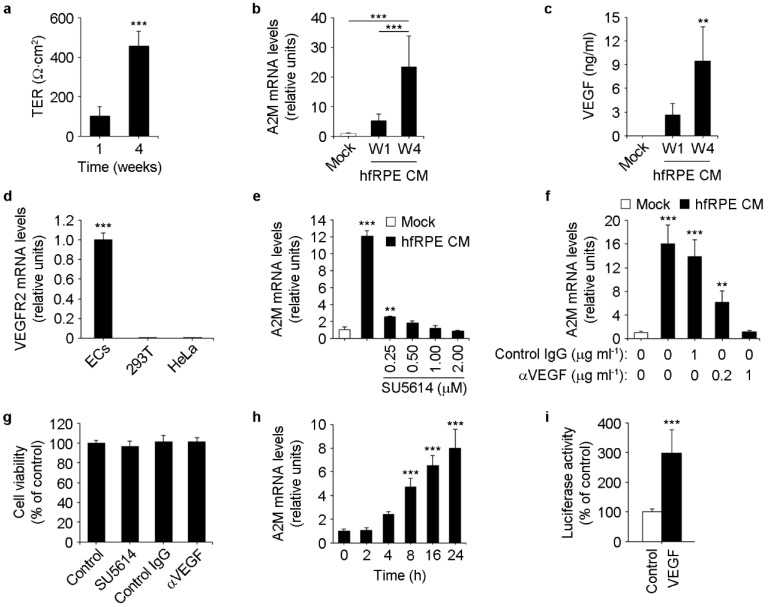
VEGF-A-dependent induction of A2M expression in ECs by RPE conditioned medium. (**a**–**c**) Positive correlation between hfRPE TER, VEGF-A levels in hfRPE basolateral conditioned media (CM) and its ability to induce A2M expression in ECs. After 1 or 4 weeks in culture (W1 and W4, respectively), hfRPE TER was measured ((**a**), *n* = 6, *t*-test) and basolateral conditioned media were collected and used to induce ECs, which were tested for A2M expression by real-time PCR 24 h after induction ((**b**), *n* = 6, ANOVA). Collected media were also used for the assessment of VEGF-A levels by ELISA ((**c**), *n* = 6, *t*-test). (**d**) Real-time PCR showing very low VEGR2 expression in 293T and HeLa cells compared to ECs (*n* = 3, ANOVA). (**e**,**f**) The VEGFR2 inhibitor SU5614 (**e**) and a VEGF-A blocking antibody (**f**) inhibit the induction of A2M expression in ECs by hfRPE conditioned medium (24 h) in a dose-dependent manner ((**e**), *n* = 3, ANOVA; *** and **, vs. 0 µM SU5614; (**f**), *n* = 5, ANOVA; *** and **, vs. mock). (**g**) Cell viability of ECs after a 24 h exposure to RPE conditioned medium in the absence (control) or presence of 2 µM SU5614, 1 µg mL^−1^ control or anti-VEGF-A antibody (control, *n* = 9; rest, *n* = 6; ANOVA). (**h**) Real-time PCR showing A2M mRNA levels in ECs after incubation with recombinant VEGF-A for different times (*n* = 6, ANOVA). ***, 0 h vs. 8 h, 16 h and 24 h. (**i**) Luciferase assays showing transcriptional activation of human *A2M* promoter after incubation of transiently transfected ECs with recombinant VEGF-A for 8 h (*n* = 6, *t*-test).

Next, we hypothesized that full maturation of RPE cells might be a requirement for them to secrete the factors needed to induce A2M expression in choroid ECs. In vitro, hfRPE maturation takes place over the course of 4–6 weeks in culture, during which transepithelial electrical resistance (TER) reaches a plateau of 500–1000 Ω cm^2^ and cells acquire RPE polarity features [26,30,31]. To test our hypothesis, we cultured hfRPE on Transwell inserts and collected basolateral conditioned media after 1 or 4 weeks in culture, when TER was ~100 and ~500 Ω cm^2^, respectively (Figure 3a). Incubation of ECs with conditioned media from 4-week hfRPE cultures induced a markedly higher increase in A2M expression than conditioned media from 1-week cultures (Figure 3b).

We next tested whether RPE-secreted vascular endothelial growth factor-A (VEGF-A) might be responsible for the induction of A2M expression in ECs, based on three pieces of evidence: (i) *VEGFA* is a human RPE signature gene, being expressed in both native and cultured RPE more than 15-fold over the levels found in other 78 tissues [32]; (ii) polarized RPE monolayers cultured on Transwell inserts secrete VEGF-A preferentially from their basolateral side [26,33], topologically consistent with a potential effect on choroid ECs in vivo; and (iii) primary porcine RPE and the RPE cell line ARPE19 increase basolateral VEGF-A secretion progressively during several weeks in culture [34,35,36]. Consistently with these reports, we found that basolateral conditioned media from 4-week RPE cultures contained significantly higher levels of VEGF-A than those found in 1-week cultures (Figure 3c). On the other hand, relative to ECs, we observed virtually no expression of the VEGF-A receptor VEGFR2 in the non-endothelial cell lines 293T and HeLa (Figure 3d), in which A2M expression does not increase in response to RPE conditioned media (Figure 2d). In summary, these results establish a positive correlation between RPE maturation in vitro, VEGF-A levels in RPE basolateral conditioned medium and its ability to induce A2M expression in VEGFR2-expressing ECs.

To directly study whether RPE-secreted VEGF-A induces A2M expression in ECs, we carried out experiments using inhibitors of the VEGF-A pathway. We incubated ECs with RPE conditioned media in the absence or presence of either the VEGFR2 inhibitor SU5614 or a VEGF-A blocking antibody, and assessed A2M expression in ECs by real-time PCR. Both treatments abolished the induction of A2M expression in a dose-dependent manner (Figure 3e,f) without compromising cell viability (Figure 3g). Moreover, we observed that recombinant VEGF-A was sufficient to induce A2M expression in ECs (Figure 3h) and to increase the transcriptional activity of human *A2M* promoter [29] in ECs transiently transfected with a luciferase-based reporter plasmid (Figure 3i). Collectively, these results demonstrate the key role of VEGF-A in RPE-mediated paracrine induction of A2M expression in ECs.

### 3.4. RPE-Induced A2M Expression in ECs Reduces MMP-2 Activity in Culture Supernatants

**Figure 4:**
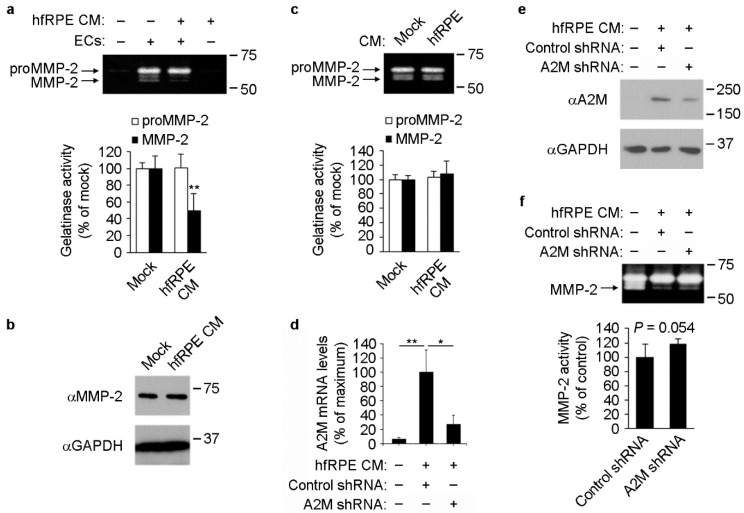
A2M-dependent inhibition of MMP-2 activity in supernatants of ECs incubated with hfRPE conditioned medium. (**a**) *Top*, gelatin zymography showing MMP-2 activity in supernatants of ECs incubated with mock or hfRPE basolateral conditioned medium (CM) (lanes 2 and 3, respectively) for 72 h. Note the absence of detectable MMP-2 activity in control medium (lane 1) and hfRPE conditioned medium that had never been in contact with ECs (lane 4). *Bottom*, quantification of proMMP-2 and MMP-2 gelatinase activity (*n* = 3, *t*-test). **, mock vs. hfRPE. (**b**) Representative Western blots (*n* = 3) showing MMP-2 and GAPDH levels in culture supernatants and lysates, respectively, of ECs after incubation of with mock or hfRPE basolateral conditioned media for 72 h. (**c**) *Top*, gelatin zymography showing MMP-2 activity in supernatants collected from ECs and subsequently incubated for 1 h at 37 °C in a test tube together with mock or hfRPE conditioned medium. *Bottom*, quantification of proMMP-2 and MMP-2 gelatinase activity (*n* = 3, *t*-test). (**d**,**e**) A2M knockdown in ECs. Real time PCR showing A2M mRNA levels (**d**) (*n* = 3, ANOVA; **, first vs. second bar; *, second vs. third bar) and Western blots showing A2M and GAPDH levels in culture supernatants and lysates, respectively (**e**), of ECs after shRNA-mediated A2M knockdown and incubation with mock or hfRPE conditioned media for 72 h. (**f**) *Top*, gelatin zymography showing MMP-2 activity in supernatants of untransduced (lane 1), control shRNA- (lane 2) and A2M shRNA-expressing (lane 3) ECs after incubation with mock (lane 1) or hfRPE conditioned medium (lanes 2 and 3) for 72 h. *Bottom*, quantification of MMP-2 gelatinase activity (*n* = 6, *t*-test).

A main feature of A2M is its ability to inhibit a wide array of proteases [15,18,19]. Thus, we studied whether RPE-mediated increase in A2M expression and secretion by ECs resulted in decreased protease activity of EC supernatants. After incubation of ECs with mock or RPE conditioned media, culture supernatants were collected and used to carry out gelatin zymography assays to analyze matrix metalloproteinase 2 (MMP-2) protease activity. Cultured ECs abundantly secrete the inactive MMP-2 form (proMMP-2) [37,38,39], which can be subsequently cleaved on the cell surface by MT1-MMP rendering active MMP-2 [40], which in turn has been described to be inhibited by A2M [16,17]. Although proMMP-2 is inactive in its original conformation, it is well established that it displays gelatinolytic activity after its denaturalization and subsequent in-gel re-naturalization that takes place during the assay [41,42]. We did not detect changes in proMMP-2 activity, suggesting that the levels of secreted proMMP-2 were not affected by exposure of ECs to RPE conditioned medium (Figure 4a). This observation was further confirmed by Western blot assays, which showed no changes in MMP-2 protein levels in EC culture supernatants (Figure 4b). MMP-2 activity was barely detectable in mock or RPE basolateral conditioned medium that had never been in contact with ECs (Figure 4a, lanes 1 and 4, respectively). However, supernatants from ECs contained clearly detectable levels of MMP-2 gelatinase activity (Figure 4a, lane 2). Importantly, supernatants from ECs that had been exposed to RPE conditioned medium had significantly lower MMP-2 activity than mock-treated cells (Figure 4a, lane 3 vs. 2). No inhibitory effect of RPE conditioned medium on MMP-2 activity was detected using a cell-free system, where RPE conditioned media were incubated with EC culture supernatants in test tubes and subsequently MMP-2 activity in the mix was assessed by gelatin zymography (Figure 4c). This result strongly suggests that RPE basolateral conditioned medium per se is not able to inhibit MMP-2 present in EC culture supernatants. Rather, our data show that RPE conditioned medium must be in contact with ECs to promote the inhibition of MMP-2 in culture supernatants, suggesting that RPE-mediated induction of A2M expression in ECs may be the mechanism of MMP-2 inhibition. 

To directly test this hypothesis, we knocked down A2M in ECs and exposed them to RPE-conditioned media (Figure 4d,e). After 72 h, we collected EC culture supernatants and tested MMP-2 activity by gelatin zymography. We observed increased MMP-2 activity when A2M was knocked down in ECs exposed to RPE-conditioned medium (Figure 4f). In summary, our results demonstrate that incubation of ECs with RPE conditioned medium results in reduced levels of active MMP-2 in culture supernatants, which may be due in part to RPE-mediated induction of A2M expression in ECs.

## 4. Discussion

Our results demonstrate that RPE-secreted VEGF-A induces A2M expression in ECs in vitro. Because *VEGFA* is expressed at high levels in RPE in vivo [32], this may account for the enrichment in A2M expression in choroidal ECs shown by scRNAseq analysis of human RPE/choroid. Although RPE-secreted VEGF-A is known to regulate choriocapillaris survival [5,6,43] and maturation of choroid EC fenestrations in rats and mice [44,45], its possible roles in the activation of choroid EC genes with the potential to regulate choroidal homeostasis remain largely unknown. Our demonstration that RPE-secreted VEGF-A induces EC expression of A2M, a key regulator of protease activity and ECM turnover, opens important questions for the future, such as how disruption of this crosstalk may affect choroidal homeostasis, RPE viability and retinal function. 

In humans, the presence of A2M in the RPE/choroid has been demonstrated by immunocytochemistry [46] and proteomics studies [13,47,48]. Microarray and bulk RNAseq analyses showed local A2M expression in the RPE/choroid [12,49,50] with some evidence of enrichment at the macular region [49,50], where A2M RNA levels are ~10-fold higher than in the neural retina [50]. However, because in humans A2M is produced by several tissues and its blood concentration is high (~2–4 g/L) [18], the relative contribution of systemic and locally expressed A2M to choroidal A2M levels are difficult to evaluate. Nevertheless, circumstantial evidence suggests that local expression is a major determinant of choroidal and Bruch’s membrane A2M levels. Given A2M high molecular weight (720 kDa) and its estimated Stokes radius of ~9 nm [51], circulating A2M cannot freely diffuse out from the choriocapillaris across endothelial tight junctions or fenestral diaphragms, which are permeable to molecules with Stokes radius < 3.2 nm [52,53]. This scenario is well documented in joints, where subsynovial capillaries are also fenestrated and A2M concentration in the synovial fluid is ~20-fold less than in serum [54]. Alternatively, A2M could reach Bruch’s membrane from circulation by transcytosis across choroidal ECs, which is the case for albumin [55]. However, A2M endocytosis is receptor-mediated, since it requires the exposure of the LRP1-binding domain which only takes place after A2M conformational change induced by protease cleavage [15,19]. Whereas A2M in its native, non-cleaved state remains in the circulation for hours, cleaved A2M is rapidly cleared from circulation (t_1/2_ 2–5 min) and degraded mostly in the liver [18,56,57,58]. Therefore, almost all circulating A2M is in its non-cleaved state, with the LRP1 binding domain unexposed, thus incapable of being endocytosed. Therefore, it is unlikely that circulating A2M can exit choroidal blood vessels by receptor-mediated transcytosis. Fluid-phase transcytosis is also unlikely, since exogenous catalase, which has a smaller Stokes radius than A2M (5.2 nm), cannot be detected in Bruch’s membrane or RPE up to 4 h after intravenous injection [52]. Noteworthy, because A2M expression can be induced in different cell types by a variety of stimuli, it has been proposed that local regulation of A2M levels may be independent of plasma A2M concentration [19]. Similarly, several studies have shown that the absence or mutation of certain serum proteins, such as matrix Gla protein (MGP) and complement factor H (CFH), induce tissue-specific phenotypes that cannot be rescued by increasing their systemic levels [59,60,61]. Moreover, it was recently reported that RPE-specific *Vegfa* deletion reduces CFH mRNA and protein levels in RPE/choroid [62], showing that local regulation of choroidal gene expression can be determinant for the tissue-specific abundance of soluble proteins that have a high concentration in serum. In summary, regulation of choroidal A2M expression may significantly contribute to local protein levels, and the potential alteration of such regulation during different ocular pathologies deserves further study.

Our studies have important implications for choroid physiopathology and for the pathogenesis of AMD. A key feature of A2M is its ability to inhibit a wide array of proteases, including ECM-remodeling MMPs. Of note, MMP-2 and -9 synergize to promote choroidal neovascularization [63], and both proteins can be inhibited by A2M [16,17]. Moreover, deletion of *Timp3*, an MMP inhibitor, results in altered choroidal vasculature in mice [64]. In addition, several ECM-related genes have been directly associated with AMD [9,10,11]. These studies strongly suggest that maintenance of choroidal ECM homeostasis is critical for retinal function, and therefore A2M could participate in such regulation. In early AMD there is choriocapillaris loss [65], which may result in reduced A2M production. Even in the absence of choriocapillaris loss, the accumulation of drusen during early AMD or Bruch’s membrane thickening during aging could impair RPE-secreted VEGF-A to reach the choriocapillaris, resulting in decreased A2M expression and therefore increasing protease activity in the choroid and Bruch’s membrane. This could pave the way for AMD progression with subsequent choroidal neovascularization through a more fragile ECM. On the other hand, A2M is a potent inhibitor of tryptase [66], a protease expressed by human choroidal mast cells [67]. Notably, AMD patients show an increased accumulation of total and degranulated mast cells compared to age-matched controls [68,69]. Thus, A2M may also be involved in the regulation of mast cell-mediated responses in the choroid. Moreover, A2M conformational change after protease cleavage not only allows A2M to inhibit the protease, but also to act as a sensor of increased proteolysis and trigger signals to counteract injury or inflammation [15]. Indeed, interaction of cleaved A2M with macrophage-expressed LRP1 modulates the inflammatory response [70,71]. Furthermore, A2M can bind and modulate the availability or/and activity of cytokines and growth factors including bFGF, PDGF, TGFβ, IL6, TNFα and VEGF-A, highlighting its potential to regulate tissue homeostasis and immunomodulation [14,72,73]. In addition, A2M interacts with β-amyloid peptide inhibiting fibril formation [74,75] and promoting β-amyloid clearance from the brain [76]. Because AMD is directly related to RPE/choroid inflammation [8,12,77] and β-amyloid accumulation [78,79], it would be very interesting to study whether choroidal A2M regulates these processes. In summary, the marked overlap between A2M functions and the molecular pathways involved in AMD onset and progression suggests an important role of A2M in maintaining choroidal homeostasis and retinal function. Interestingly, bulk RNAseq studies showed a significant decrease in A2M expression in human RPE/choroid ex vivo after treatment with C-reactive protein [80], which is elevated in the serum of late AMD patients [81] and accumulates in the choriocapillaris and Bruch’s membrane of CFH high-risk AMD donors [80]. Moreover, treatment of neovascular AMD patients with VEGF-A inhibitors could also reduce choroidal A2M levels, which may contribute to some of the side effects associated with such therapy [82]. Further studies are needed to elucidate whether choroidal A2M expression and/or function are altered in different stages of AMD and to determine their potential as new therapeutic targets. 

## Data Availability

Human RPE/choroid scRNAseq analysis was carried out with published data [20,21,22,23,24] using Spectacle software [25] (https://singlecell-eye.org/, v0.3.1, accessed on 29 July 2021). The rest of the data presented in this study are available on request from the corresponding author.

## Supplementary Materials

The following supporting information can be downloaded at: https://www.mdpi.com/article/10.3390/cells11192975/s1, Figure S1: original, unmodified, and uncropped images from all Western blots presented; Figure S2: original, unmodified, and uncropped images from all zymography assays presented.
