# Supplementary figures and images for: Retinal Pigment Epithelium-Secreted VEGF-A Induces Alpha-2-Macroglobulin Expression in Endothelial Cells
Source: Cells. 2022 Sep 24;11(19):2975. doi: 10.3390/cells11192975 (PMC9564307; doi:10.3390/cells11192975)

Figure 2b, αA2M

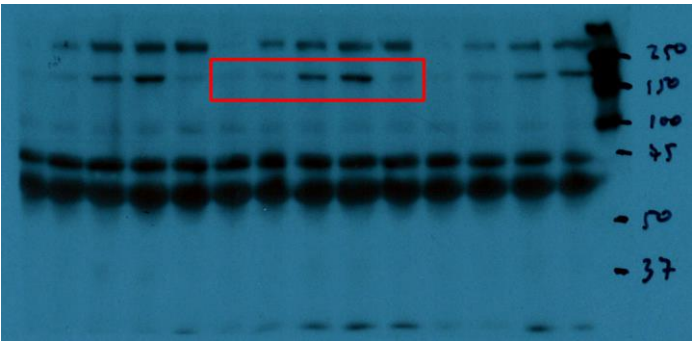

Figure 2b, αGAPDH

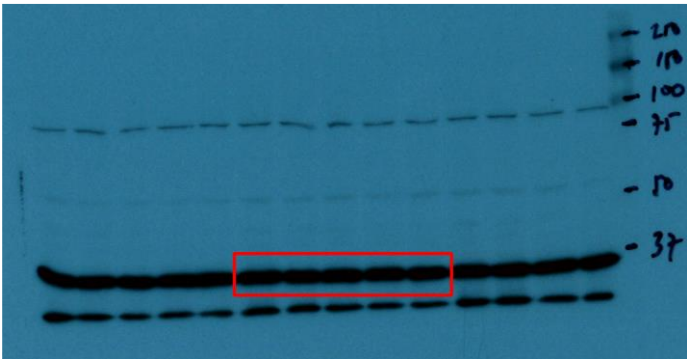

Figure 4b, αMMP-2

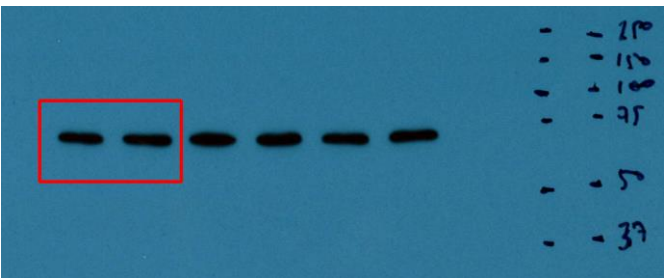

Figure 4b, αGAPDH

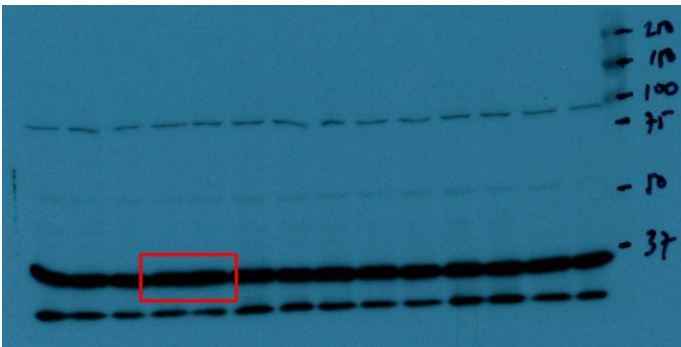

Figure 4e, αA2M

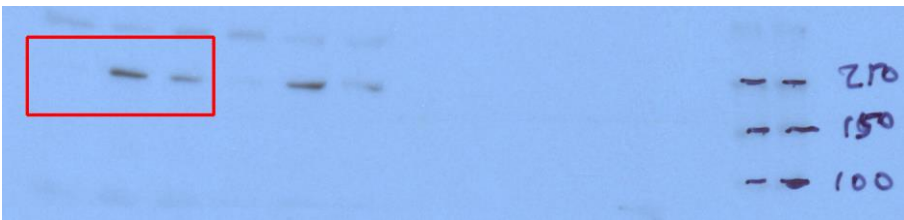

Figure 4e, αGAPDH

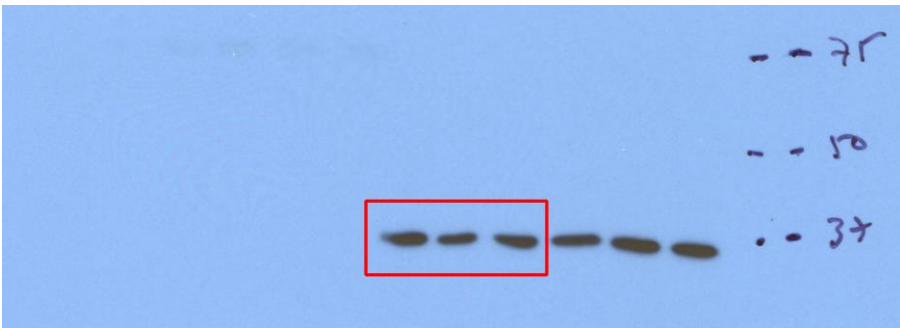

Supplement: Supplementary file 1 [file cells-11-02975-s001.zip › Lehmann et al Supplementary Figure S1.pdf]

Figure 4a

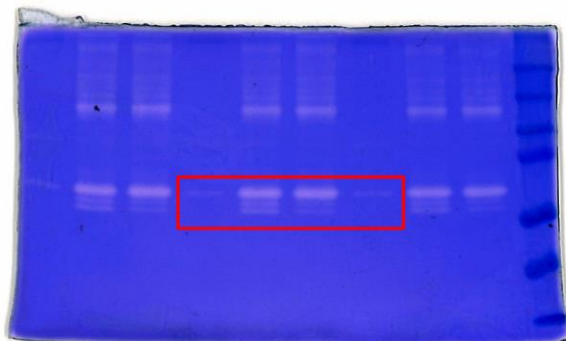

Figure 4c

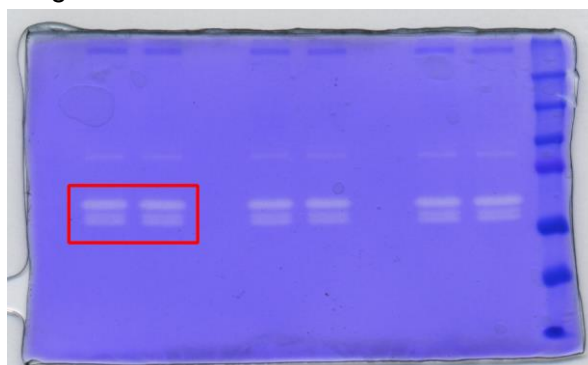

Figure 4f

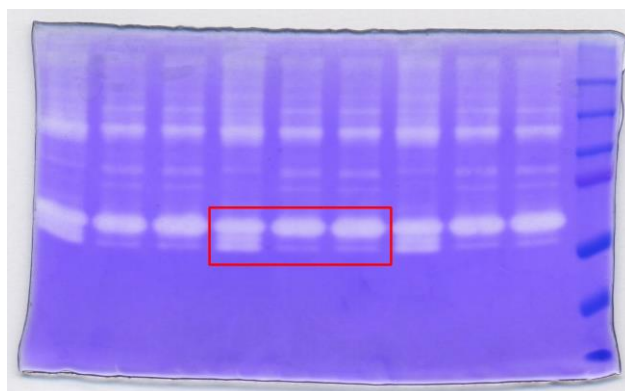

Supplement: Supplementary file 1 [file cells-11-02975-s001.zip › Lehmann et al Supplementary Figure S2.pdf]
